# MultiLoc2: integrating phylogeny and Gene Ontology terms improves subcellular protein localization prediction

**DOI:** 10.1186/1471-2105-10-274

**Published:** 2009-09-01

**Authors:** Torsten Blum, Sebastian Briesemeister, Oliver Kohlbacher

**Affiliations:** 1 Division for Simulation of Biological Systems, ZBIT/WSI, Eberhard-Karls-Universität Tübingen, Germany

## Abstract

**Background:**

Knowledge of subcellular localization of proteins is crucial to proteomics, drug target discovery and systems biology since localization and biological function are highly correlated. In recent years, numerous computational prediction methods have been developed. Nevertheless, there is still a need for prediction methods that show more robustness and higher accuracy.

**Results:**

We extended our previous MultiLoc predictor by incorporating phylogenetic profiles and Gene Ontology terms. Two different datasets were used for training the system, resulting in two versions of this high-accuracy prediction method. One version is specialized for globular proteins and predicts up to five localizations, whereas a second version covers all eleven main eukaryotic subcellular localizations. In a benchmark study with five localizations, MultiLoc2 performs considerably better than other methods for animal and plant proteins and comparably for fungal proteins. Furthermore, MultiLoc2 performs clearly better when using a second dataset that extends the benchmark study to all eleven main eukaryotic subcellular localizations.

**Conclusion:**

MultiLoc2 is an extensive high-performance subcellular protein localization prediction system. By incorporating phylogenetic profiles and Gene Ontology terms MultiLoc2 yields higher accuracies compared to its previous version. Moreover, it outperforms other prediction systems in two benchmarks studies. MultiLoc2 is available as user-friendly and free web-service, available at: .

## Background

A eukaryotic cell is organized into different membrane-surrounded compartments which are specialized for different cellular functions. However, most cellular proteins are synthesized in the cytoplasm and need to be transported to their final location to fulfill their biological function. The whole protein sorting process is not completely understood but, in principle, it depends on signals in the amino acid sequence or signal patches on the protein surface.

There are diverse applications for the knowledge of the localization of the complete proteome, the localizome, in the fields of proteomics, drug target discovery and systems biology. Since subcellular localization is highly correlated with biological function, it is possible to draw conclusions from the knowledge of a protein's localization regarding its cellular role. Hence, subcellular localization is a key functional characteristic of proteins. Proteins destined for the extracellular space or the cell surface are especially of pharmaceutical interest as they are easily accessible drug targets. The integration of large-scale localization data with diverse omics data, produced by high-throughput techniques, will help in understanding cellular function. Localization data can be used to validate or analyze protein-protein interactions inferred from two-hybrid experiments or biochemical pathways inferred from microarray expression data.

In recent years large-scale sequencing projects have caused a rapid growth of sequence information and increased the number of proteins but without any further annotation in public databases. Determining the localization of proteins using experimental methods alone is expensive and time-consuming.

Fast and accurate computational prediction methods provide an attractive complement to experimental methods. In the last decade numerous computational methods, which can be roughly divided into sequence-based and annotation-based methods [1,2], have been developed. Sequence-based predictors only use the amino acid sequence of the query protein as input. They are based either on the detection of sequence-coded sorting signals like N-terminal targeting peptides [3-11] and nuclear localization signals (NLS) [11] or use the fact that the amino acid composition of a protein is correlated with its localization [12]. The latter methods [2,13-21] use different kinds of composition information like the overall, paired, gapped-paired, surface or pseudo amino acid composition from the protein sequence or sequence profiles. More recent and advanced methods combine composition information with the detection of sorting signals [22,23]. Annotation-based predictors search the sequence for functional domains and motifs [24,25] or use textual information like Swiss-Prot keywords [26,27], Gene Ontology (GO) terms [28,29] or PubMed abstracts [30,31]. If such information is not available for the query protein most of these methods transfer annotation from close homologs. Nair and Rost [32] quantitatively showed that proteins with sufficiently similar sequences usually are close homologs that function at the same localization site. Annotation-based predictors often report higher performance than sequence-based predictors which, however, are more general and robust and can also be used for novel proteins for which no additional information is present and no annotated close homologs can be found. In addition to the predictors of the two categories, there are also hybrid approaches which combine sequence-based and annotation-based information [33-37] and can therefore profit from the advantages of both worlds. A further category are meta predictors, which integrate the prediction results of multiple tools [38,39].

Although there already exist a lot of computational prediction methods, there is still room for improvement. This is due to the fact that the protein sorting process is very complex and not yet well understood. Only a small portion of proteins have clearly identifiable sorting signals in their primary sequence. As a consequence, available prediction methods are often either specialized for the prediction of very few localizations with higher accuracy or for the prediction of a wide range of localizations with reduced accuracy. A further challenge is how to deal with proteins present in multiple locations [40,41].

The aim of this work was not to develop a completely novel algorithm or protein coding but to create a reliable and efficient predictor with maximum prediction accuracy. Especially, in the context of large-scale genome annotations scientific users rely on high-accuracy subcellular localization predictions. A difference of a few percent points in performance can mean hundreds of correctly or incorrectly classified proteins in genome annotation. Hence, well-tuned predictors can make a significant difference in this area. To this end, we extend our previously published support vector machine (SVM) based predictor MultiLoc [23], which utilizes overall amino acid composition and the presence of known sorting signals. We show that the performance of MultiLoc can be clearly improved by incorporating phylogenetic profiles and GO terms inferred from the primary sequence leading to a high-accuracy prediction system that covers all main eukaryotic subcellular localizations. Phylogenetic profiles encode evolutionary information in the form of patterns of protein inheritance among the species. Marcotte *et al*. [42] originally applied this approach to distinguish mitochondrial and non-mitochondrial proteins. GO terms were previously combined with sequence-based information in the form of pseudo amino acid composition in a group of predictors [36,37].

The GO terms are used as primary prediction criteria and pseudo amino acid composition is used if no GO term can be found. Our extensive MultiLoc2 prediction system integrates composition and sorting signal information with phylogenetic profiles and GO terms towards a common localization prediction. The extended MultiLoc system is trained on two different datasets resulting in two versions with different resolutions. MultiLoc2-LowRes is a low resolution predictor that is specialized for globular proteins and predicts up to five localizations for animals, fungi and plants. MultiLoc2-HighRes is a high resolution predictor that covers all 11 main eukaryotic subcellular localizations. The main reason for creating MultiLoc2-LowRes additionally to MultiLoc2-HighRes is to provide a predictor with superior prediction accuracy for globular proteins. For certain applications it is sufficient to discriminate between secreted proteins and the localizations of globular proteins.

The MultiLoc2 approach was compared with current state-of-the-art tools (BaCelLo [19], LOCtree [2], Protein Prowler [9], TargetP [5] and WoLF PSORT [22]) using independent datasets sharing very low sequence identity with the training datasets of all compared tools. We found MultiLoc2 to perform considerably better than related tools for animals and plants and comparable for fungal proteins in a benchmark study with five localizations. Since GO terms are not always available, we evaluate MultiLoc2 as purely sequence-based and found the performance only slightly reduced but still better or comparable with other tools. Furthermore, MultiLoc2-HighRes performs clearly better compared with WoLF PSORT using a second independent dataset that extends the benchmark study to all main eukaryotic subcellular localizations. The second benchmark study was performed only between these two predictors since the remaining methods are specialized for a smaller amount of localizations. Both versions of MultiLoc2 are available online as web interface at . The online version provides fast access to MultiLoc2 for a limited number of query sequences. Furthermore, a stand-alone version (including the source code of the method) is available from the website. The stand-alone version is suitable for large-scale offline prediction jobs.

In the following sections the MultiLoc2 system is described in detail together with the training and test datasets used, followed by the performance evaluation and the results of the benchmark studies.

## Implementation

### MultiLoc2 architecture

**Figure 1:**
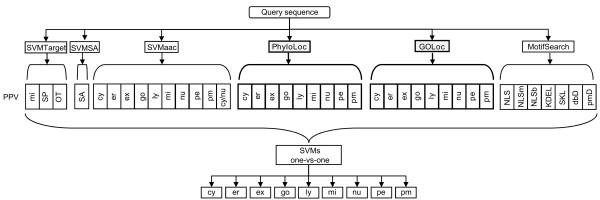
**MultiLoc2 architecture**. The architecture of MultiLoc2-HighRes (animal version). A query sequence is processed by a first layer of six subprediction methods (SVMTarget, SVMSA, SVMaac, PhyloLoc, GOLoc and MotifSearch). The two new subprediction methods, PhyloLoc and GOLoc, are highlighted in bold. The individual output of the methods of the first layer are collected in the protein profile vector (PPV), which enters a second layer of SVMs producing probability estimates for each localization.

The MultiLoc prediction system described earlier [23] is based on the integration of the output of four sequence-based subclassifiers (SVMTarget, SVMSA, SVMaac and MotifSearch) into a protein profile vector. The subclassifiers utilize the overall amino acid composition or search for specific sorting signals. MultiLoc2 extends the original architecture with two new classifiers based on phylogenetic profiles (PhyloLoc) and GO terms (GOLoc). As stated in the introduction, there are two versions of MultiLoc2 which differ in the number of predictable localizations. MultiLoc2-HighRes can deal with nuclear (nu), cytoplasmic (cy), mitochondrial (mi), chloroplast (ch), extracellular (ex), plasma membrane (pm), peroxisomal (pe), endoplasmic reticulum (er), Golgi apparatus (go), lysosomal (ly) and vacuolar (va) proteins. MultiLoc2-LowRes is specialized for globular proteins and predicts secretory pathway (SP) proteins (separated into the six classes ex, pm, er, go, ly, va in MultiLoc2-HighRes) as well as nu, cy, mi and ch. Similar to its previous version, MultiLoc2 is available for plant, animal and fungal protein localization prediction. A scheme of the overall architecture of MultiLoc2 is shown in Fig. 1. A query sequence is processed by a first layer of six subprediction methods. The results from these methods are collected in the protein profile vector, which is used as input for the final layer of SVMs, which in turn outputs the final localization prediction. In both layers one-vs-one SVMs are used for classification. The corresponding figure of MultiLoc2-LowRes is also available [see Additional file 1]. The original four sequence-based classifiers are briefly described in the next section, followed by details of PhyloLoc and GOLoc.

### Subprediction methods

#### SVMTarget

SVMTarget is based on the detection of N-terminal targeting peptides to predict ch, mi, SP and other (OT) localizations for plant proteins and only mi, SP and OT for animal and fungal proteins. A sliding window approach scans the N-terminal part of a given query sequence. The partial amino acid composition in the window is used as input for the SVMs. The output of SVMTarget is a probability for each localization.

#### SVMSA

SVMSA scans the sequence for a signal anchor (SA) which can be present in membrane proteins of the secretory pathway instead of a signal peptide. Therefore, SVMSA complements SVMTarget. SAs are also detected using a sliding window approach based on partial amino acid composition. SVMSA is specialized for membrane proteins and is therefore not included in MultiLoc2-LowRes.

#### SVMaac

SVMaac is based on the overall amino acid composition of the query sequence and outputs a probability for each localization. In contrast to the original MultiLoc, the binary one-versus-all classification is replaced by a one-versus-one procedure since a slightly performance increase could be achieved.

#### MotifSearch

MotifSearch outputs five binary features that encode the presence or absence of sequence motifs relevant to protein sorting such as nuclear localization signals (NLSs). Two additional binary features represent the presence or absence of a DNA-binding domain or a plasma membrane receptor domain.

#### PhyloLoc

**Figure 2:**
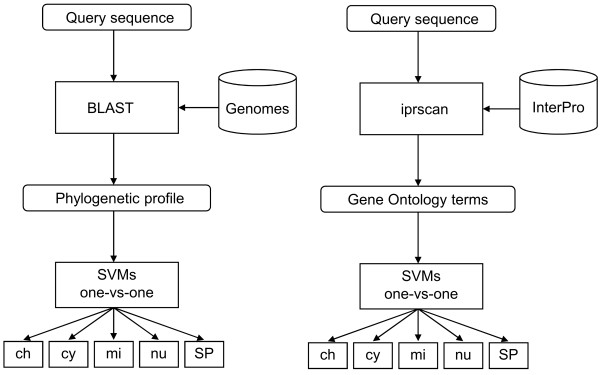
**PhyloLoc and GOLoc architecture**. The architectures of PhyloLoc and GOLoc from MultiLoc2-LowRes. The input of PhyloLoc is a vector of similarities (phylogenetic profile) between the query sequence and the best sequence match in each genome inferred from BLAST. The input of GOLoc is a binary-coded vector representing the GO terms of the query sequence inferred from InterPro using InterProScan. PhyloLoc and GOLoc use one-versus-one SVMs to process their input and to calculate probability estimates for each localization.

Proteins within the same subcellular localization tend to share a similar taxonomic distribution of homologous proteins in other genomes [42]. This kind of information can be represented as a profile [43] which encodes the pattern of presence or absence of a given protein in a set of genomes. Marcotte *et al*. [42] applied phylogenetic profiles for the distinction of mitochondrial and non-mitochondrial proteins using 31 genomes and a linear discrimination function. PhyloLoc is based on phylogenetic profiles derived from 78 fully sequenced genomes and SVMs to predict all of the localizations of the MultiLoc2 predictors. The genomes were retrieved from the National Center for Biotechnology Information (NCBI) web site (downloaded between 6th and 9th February 2008). We used all available eukaryotic (20) and archaean (33) genomes and a non-redundant set of 25 bacterial genomes [see Additional file 1]. The input of PhyloLoc (as shown in Fig. 2) is a vector of similarities between the query sequence and the best sequence match in each genome using BLAST. The BLAST homology searches are performed using default settings. The bit score *B*_*qi *_of the best sequence match of the query sequence *q *in genome *i *and the self bit score *B*_*qq *_of *q *aligned with itself are used to calculate the similarity *S*_*qi *_which is defined as: *S*_*qi *_= *B*_*qi*_/*B*_*qq*_. Due to the fact that *B*_*qi *_is always smaller than *B*_*qq*_, the values of *S*_*qi *_range from zero to one. Values close to one indicate presence of the query protein and values close to zero indicate absence. The calculation of phylogenetic profiles based on bit scores was also previously used for the functional annotation of bacterial genomes [44]. An important point to note is that, although BLAST is used, creating phylogenetic profiles is not an annotation-based or homology-based method as sometimes described in the literature. The reason is that there is no annotation-transfer from the aligned sequences. Actually, it is irrelevant whether the proteins of the genomes are annotated or not. Proteins with similar phylogenetic profiles are co-inherited and do not have to be close homologs [45].

#### GOLoc

The Gene Ontology (GO) is a controlled vocabulary for uniformly describing gene products in terms of biological processes, cellular components and molecular function across all organisms [46]. It has been shown that GO terms can be used to improve the performance of subcellular protein localization prediction methods [47,48]. In the literature to date, there are three possibilities for obtaining GO annotation terms for a query sequence. If the UniProt [49] accession number is known, one can simply extract the GO annotation from the UniProt database [50]. However, this procedure fails for novel proteins without accession number. Another possibility is to search for homologous proteins annotated with GO terms using BLAST [28,29]. This becomes difficult in cases where proteins have no close homolog or proteins have many homologs, because no GO term can be obtained or GO terms might be ambiguous. A further method of inferring GO terms is InterProScan [51] used, for example, by Chou and Cai [52]. Given a protein sequence, the tool scans against various pattern and signature data sources collected by the InterPro project [53]. InterPro also provides a mapping of the detected protein domains and functional sites to GO terms.

Our subpredictor GOLoc is based on GO terms calculated using InterProScan. Since the GO terms are derived directly from the query sequence, we avoid the drawbacks of using accession numbers or BLAST. The input of GOLoc is a binary-coded vector which represents all GO terms of the training sequences (see Fig. 2). GO terms present in the query sequence are set to 1 in the vector and to 0 otherwise [see Additional file 1].

### Datasets

#### BaCelLo

The datasets used for training and testing MultiLoc2-LowRes against comparable predictors were obtained from the BaCelLo website. The homology-reduced training dataset was extracted from Swiss-Prot release 48 and contains 2597 animal, 1198 fungal and 491 plant proteins resulting in three kingdom-specific predictors. By ignoring proteins annotated as 'membrane' or 'transmembrane', only globular proteins were considered.

The animal and fungal proteins represent four localizations (nu, cy, mi, SP) and the plant proteins five localizations (with the addition of ch). The independent test dataset (BaCelLo IDS) was extracted from Swiss-Prot release 54. Only proteins added to the database starting from release 49 were considered. Furthermore, proteins sharing a sequence identity >30% to at least one protein from release 48 were removed. This ensured that all test proteins were novel to the predictors in the benchmark study since all of them were trained using Swiss-Prot proteins up to release 48. In order to avoid a bias towards the prediction of over-represented protein classes, all sequences which share the same localization and a sequence identity >30% were clustered into 432 animal, 418 fungi and 132 plant groups. More information concerning the creation of the BaCelLo data sets can be found in Pierleoni *et al *[19] and Casadio *et al*. [54].

#### Höglund

For training MultiLoc2-HighRes we applied the original dataset used to train MultiLoc [23], which contains 5959 eukaryotic proteins extracted from Swiss-Prot release 42. The data set covers 11 localizations (cy, ch, er, ex, go, ly, mi, nu, pe, pm, va). To also compare the prediction performance of MultiLoc2 with WoLF PSORT in regard to the localizations not present in the BaCelLo test dataset, we created a second independent dataset (Höoglund IDS) which covers seven localizations (er, ex, go, ly, pe, pm, va). Therefore, animal, fungal and plant proteins of these localizations were extracted from Swiss-Prot release 55.3 in the same way as the BaCelLo independent dataset. However, in the case of the plant proteins, we increased the allowed sequence identity threshold to 40% in order to obtain enough data. We used BLASTClust to cluster the sequences using 30% pairwise sequence identity for the animal and fungal proteins and 40% for the plant proteins. The whole procedure delivered 158 animal, 106 fungi and 30 plant groups.

For each training dataset (BacelLo and Höglund), a table that shows the number of protein sequences for each localization is listed elsewhere [see Additional file 1]. Two different datasets were used for training since this allows a more objective performance comparison between MultLoc2-HighRes and its predecessor MultiLoc on the one hand and in particular between MultLoc2-LowRes and further methods on the other hand.

### SVM training and performance evaluation

All building blocks (except MotifSearch) of MultiLoc2 were trained using SVMs [55] from the LIBSVM [56] software. Throughout, we used the radial basis kernel function and optimized the *c *and *g *parameters by grid search. Furthermore, we defined weights for each class in order to reduce the over-prediction effect when using unbalanced training datasets. To this end, we used built-in functionality of LIBSVM. The weighting scheme assigns weight 1.0 to the largest class and higher weights to the remaining classes. The weights of these classes are simply calculated by dividing the size of the largest class by that of each smaller class. The probability estimates calculated by LIBSVM were used for ranking the final predicted localizations and choosing the most probable one.

We used five-fold cross-validation for training and evaluating the prediction performance. Additionally, independent datasets were used for testing MultiLoc2 and comparison with other prediction methods. Therefore, all test proteins share low sequence similarity with proteins in the training datasets.

Localization-specific performance results were expressed using sensitivity (SE), specificity (SP) and the Matthews correlation coefficient (MCC) defined as:



To evaluate the overall prediction performance, we used average sensitivity (AVG), which is also known as the average localization-specific accuracy, as primary measure. The average sensitivity is better suited than the overall accuracy (ACC), the percentage of correctly predicted proteins of all localizations. The reason is that all prediction methods are trained on unbalanced datasets with strongly varying numbers of proteins per localization. This often biases the prediction towards the localization with the most representations in the training dataset. Hence an unbalanced test dataset would also normally lead to a distorted performance evaluation when using the ACC only. To calculate the performance measures for the independent datasets, we used the average rates of true and false predicted proteins within each cluster.

## Results and discussion

### Cross-validation performance

The impact of the MultiLoc2 extensions on the overall prediction performance was evaluated using 5-fold cross-validation. The results are summarized in Table 1. The average sensitivity and overall accuracy of MultiLoc2-LowRes (trained on the BaCelLo dataset) and MultiLoc2-HighRes (trained on the Höglund dataset) are compared with those of the original MultiLoc architecture and MultiLoc extended by PhyloLoc as well as GOLoc only. Using the BaCelLo dataset, MultiLoc2-LowRes yields a clearly higher AVG (85.0% for animals, 83.9% for fungi and 81.6% for plants) than the original MultiLoc (77.3%, 78.4% and 71.4% respectively). For the Höglund dataset the AVG is increased from 78.6% to 89.2% for animal, from 78.0% to 89.2% for fungal and from 78.0% to 89.4% for plant proteins by the MultiLoc2-HighRes system compared to the original MultiLoc. Note that the performance results for the original MultiLoc differ from those previously reported [23] since the SVMaac architecture has slightly changed. Adding PhyloLoc or GOLoc individually to MultiLoc already increased the performance considerably, whereas the performance gain caused by GOLoc is slightly higher compared to PhyloLoc. However, the best performance is achieved by the addition of both subpredictors, obtaining MultiLoc2. Similar trends can be detected regarding the overall accuracies. The standard deviations of the MultiLoc2-LowRes plant version are higher compared to the other versions due to the fact that the number of training sequences in the dataset is considerably lower.

**Table 1:** Cross-validation performance comparison of different MultiLoc architectures trained using the BaCelLo and the Höglund datasets

**Dataset**	**Method**	**Animals**		**Fungi**		**Plants**	
		**AVG**	**ACC**	**AVG**	**ACC**	**AVG**	**ACC**
BaCelLo							
	MultiLoc	77.3 (± 2.9)	75.7 (± 3.1)	78.4 (± 2.7)	71.0 (± 2.6)	71.4 (± 6.8)	67.8 (± 3.8)
	+ PhyloLoc	80.1 (± 2.4)	78.2 (± 2.9)	80.0 (± 2.5)	73.6 (± 0.9)	78.6 (± 3.6)	77.4 (± 1.9)
	+ GOLoc	84.0 (± 1.7)	82.8 (± 2.0)	81.1 (± 0.5)	75.5 (± 1.1)	80.9 (± 4.4)	77.6 (± 3.5)
	MultiLoc2-LowRes	86.1 (± 1.4)	84.0 (± 1.7)	82.8 (± 2.2)	77.9 (± 0.5)	81.9 (± 4.1)	80.2 (± 3.5)
Höglund							
	MultiLoc	78.6 (± 1.2)	76.4 (± 1.2)	78.0 (± 1.3)	76.6 (± 1.2)	78.0 (± 1.8)	76.4 (± 1.7)
	+ PhyloLoc	84.6 (± 0.7)	84.0 (± 0.6)	84.7 (± 1.4)	84.4 (± 0.9)	86.5 (± 1.5)	84.3 (± 0.7)
	+ GOLoc	87.3 (± 1.8)	86.7 (± 1.0)	87.1 (± 0.9)	86.9 (± 0.8)	86.9 (± 1.4)	86.3 (± 1.1)
	MultiLoc2-HighRes	89.3 (± 1.4)	88.6 (± 1.0)	89.2 (± 1.1)	88.9 (± 1.2)	89.4 (± 0.8)	88.7 (± 0.9)

This table compares the average sensitivities (AVGs) and overall accuracies (ACCs) of MultiLoc2-LowRes and MultiLoc2-HighRes with those of the original MultiLoc and the extended architecture based on PhyloLoc as well as GOLoc only. The AVGs and ACCs are given in percent. The standard deviations (in parentheses) refer to the differences of the AVGs and ACCs of the different cross-validation models.

### Comparison with related tools

In a recently published study [54] five selected top-performing sequence-based prediction methods (BaCelLo, LOCtree, Protein Prowler, TargetP and WoLF PSORT) were compared using an independent dataset (see Section 2.3.1). Based on this benchmark study, we compared the performance of MultiLoc2 against these five methods using the same test setting. The benchmark study considered five subcellular localizations (nu, cy, mi, ch, SP). Furthermore, a virtual class nu/cy, containing nu and cy proteins, was created in order to ensure a fair comparison with TargetP and Protein Prowler which do not discriminate between these two localizations. To deal with WoLF PSORT and LOCtree, predicted sublocalizations of the secretory pathway were grouped into the SP class. A similar approach was followed for the evaluation of MultiLoc2-HighRes. Depending on the inclusion of the virtual nu/cy class, the number of tested classes is three or four for animals and fungi as well as four or five for plants. We also evaluated the performance of only sequenced-based predictions of MultiLoc2 by disregarding GO terms to simulate the case of unavailability of GO terms. Table 2 shows the localization-specific performance results using sensitivity, specificity and MCC and Table 3 summarizes the overall performances using AVG and ACC. Note that the number of SP clusters for fungi (9) and plants (6) and the mi clusters for plants (6) is quite small compared to the remaining localizations. Therefore, some care should be taken when interpreting the prediction results. Small clusters have only a small influence on the ACC, however, a large influence on the AVG.

**Table 2:** Comparison of the localization-specific prediction results using BaCalLo independent dataset (BacelLo IDS)

		**Animals**				**Fungi**				**Plants**			
**Predictor**	**Loc**	**No**.	**SE**	**SP**	**MCC**	**No**.	**SE**	**SP**	**MCC**	**No**.	**SE**	**SP**	**MCC**
**MultiLoc2-LowRes**	SP	75	97	97	0.89	9	78	98	0.60	6	83	95	0.58
	mi	48	89	97	0.81	77	68	94	0.62	6	67	96	0.51
	ch	-	-	-	-	-	-	-	-	72	77	94	0.72
	nu	224	62	93	0.57	152	63	79	0.36	36	91	90	0.77
	cy	85	72	82	0.43	180	54	78	0.27	17	41	94	0.38
	nu/cy	308	93	96	0.87	332	92	78	0.63	52	94	92	0.84

**MultiLoc2-HighRes**	SP	75	87	95	0.79	9	78	98	0.63	6	83	93	0.50
	mi	48	83	96	0.75	77	51	95	0.52	6	67	93	0.40
	ch	-	-	-	-	-	-	-	-	72	53	94	0.51
	nu	224	58	93	0.54	152	50	84	0.32	36	86	91	0.74
	cy	85	71	80	0.39	180	56	75	0.22	17	37	87	0.20
	nu/cy	308	91	91	0.78	332	84	76	0.48	52	93	84	0.74

**BaCelLoc**	SP	75	93	97	0.88	9	100	98	0.74	6	100	95	0.66
	mi	48	74	95	0.66	77	79	87	0.58	6	17	100	0.40
	ch	-	-	-	-	-	-	-	-	72	71	83	0.54
	nu	224	57	83	0.41	152	72	67	0.38	36	88	78	0.60
	cy	85	51	74	0.21	180	32	84	0.19	17	27	98	0.38
	nu/cy	308	93	92	0.83	332	85	83	0.61	52	88	84	0.70

**LOCtree**	SP	75	79	91	0.65	9	78	92	0.35	6	83	96	0.60
	mi	48	64	92	0.51	77	42	92	0.38	6	58	90	0.30
	ch	-	-	-	-	-	-	-	-	72	77	88	0.66
	nu	224	66	73	0.39	152	63	59	0.22	36	72	89	0.61
	cy	85	35	86	0.22	180	35	78	0.15	17	33	97	0.39
	nu/cy	308	84	83	0.64	332	83	49	0.31	52	75	93	0.70

**Protein Prowler**	SP	75	86	99	0.88	9	93	99	0.20	6	100	92	0.61
	mi	48	51	99	0.71	77	33	99	0.51	6	67	86	0.40
	ch	-	-	-	-	-	-	-	-	72	7	95	0.40
	nu/cy	308	98	73	0.79	332	98	40	0.52	52	86	77	0.52

**TargetP**	SP	75	88	98	0.88	9	89	97	0.56	6	100	93	0.61
	mi	48	82	92	0.63	77	50	92	0.44	6	50	91	0.26
	ch	-	-	-	-	-	-	-	-	72	55	91	0.49
	nu/cy	308	89	89	0.75	332	89	59	0.48	52	83	79	0.62

**WoLF PSORT**	SP	75	92	94	0.80	9	89	99	0.73	6	33	95	0.24
	mi	48	71	95	0.63	77	53	90	0.44	6	42	99	0.52
	ch	-	-	-	-	-	-	-	-	72	61	81	0.43
	nu	224	77	81	0.58	152	93	39	0.35	36	72	83	0.52
	cy	85	34	88	0.23	180	11	98	0.19	17	24	83	0.28
	nu/cy	308	89	90	0.76	332	89	57	0.46	52	87	74	0.61

The sensitivity (SE) and specificity (SP), given in percentages, and Matthews correlation coefficient (MCC) are listed for each localization (Loc). The number of clusters (No.) per localization is also shown. In Protein Prowler and TargetP, predictions for nu and cy are only available grouped as nu/cy.

**Table 3:** Comparison of the overall performance results using BaCelLo independent dataset (BaCelLo IDS)

**Predictor**	**Classes**	**Animals**	**Fungi**	**Plants**
**MultiLoc2-LowRes**	3	**93 **(**93**)	79 (**87**)	**80 **(**83**)
	4	**80 **(**73**)	66 (**60**)	**72 **(**76**)
**MultiLoc2-HighRes**	3	87 (89)	71 (76)	74 (71)
	4	75 (68)	59 (52)	65 (62)
**BaCelLo**	3	87 (91)	**88 **(84)	69 (76)
	4	69 (64)	**71 **(57)	61 (69)
**LOCtree**	3	76 (81)	68 (75)	73 (76)
	4	61 (62)	55 (47)	65 (70)
**Protein Prowler**	3	78 (91)	75 (86)	65 (63)
	4	-	-	-
**TargetP**	3	86 (88)	76 (82)	72 (67)
	4	-	-	-
**WoLF PSORT**	3	84 (88)	77 (82)	56 (69)
	4	69 (71)	62 (51)	46 (57)

The average sensitivity and the overall accuracy (in parentheses) for the prediction of three and four classes for animals and fungi and four and five classes for plants are shown. Both measures are given in percentages. The top-scoring average sensitivity and average accuracy are highlighted in bold. Results for Protein Prowler and TargetP predictions are only available for a reduced number of classes since nu and cy are grouped as nu/cy.

MultiLoc2-LowRes always yields the highest ACCs and AVGs for animal and plant proteins and hence outperforms all other predictors. The reason for this outstanding result is that MultiLoc2-LowRes is, in general, better suited to discriminate between nu and cy and between mi and ch proteins (see Table 2), which is a known challenge in the prediction of protein subcellular localization. For fungal proteins the ACCs are the highest and the AVGs are the second highest after the BaCelLo predictor. One reason for the reduced AVG performance is that on average only 34% of the fungal proteins are annotated with GO terms by InterProScan. The annotation-rate is higher for animals (43%) and plants (79%). Compared to MultiLoc2-LowRes, the performance of MultiLoc2-HighRes is, not surprisingly, reduced, since it is a more general predictor not specialized for globular proteins and covering a wider range of localizations. However, for animal and plant proteins the AVGs of MultiLoc2-HighRes are equal or higher compared to the remaining methods. Similar to MultiLoc2-LowRes, MultiLoc2-HighRes performs worse for fungal proteins. The AVGs are still better than LOCtree, however, worse compared with Protein Prowler, TargetP and WoLF PSORT.

If we simulate the case in which no GO terms are available for any test proteins, the overall performances of the MultiLoc2 predictors are slightly reduced but still better than those of the other methods for animal and plant and comparable for fungal proteins [see Additional file 1].

In a second benchmark study, MultiLoc2-HighRes and WoLF PSORT were compared using the Höglund independent dataset (see Section 2.3.2). In contrast to the other predictors, both methods allow the prediction of all main eukaryotic subcellular localizations. We further note that WoLF PSORT can also distinguish between the cytoskeleton within the cytoplasm. In this comparison we only consider those localizations (ex, pm, pe, er, go, ly, va) not tested in the previous study. Since it is known that discriminating between these classes is challenging, we also evaluated whether the tested proteins could be correctly predicted within the top three ranked localizations. The results of this study are summarized in Table 4. MultiLoc2-HighRes always achieves clearly higher AVGs. In particular, the AVG within the top three locations of MultiLoc2 is about twice as high as that of WoLF PSORT. A similar result is observed regarding the ACCs. MultiLoc2-HighRes has a much lower bias towards overrepresented localizations and, thus, almost never shows zero sensitivity for a localization with few representatives. This again proves high robustness of MultiLoc2, even in cases of many localizations.

**Table 4:** Performance comparison of MultiLoc2-HighRes with WoLF PSORT using Höglund independent dataset (Höglund IDS)

		**Animals**			**Fungi**			**Plants**		
**Predictor**	**Loc**	**No**.	**SE**	**SE3**	**No**.	**SE**	**SE3**	**No**.	**SE**	**SE3**
**MultiLoc2-HighRes**	ex	78	78	91	7	77	86	1	0	100
	pm	34	55	78	29	10	31	6	33	50
	pe	3	33	100	5	20	100	2	50	100
	er	25	28	70	46	46	83	6	50	83
	go	14	7	57	8	25	63	6	33	50
	ly	4	25	75	-	-	-	-	-	-
	va	-	-	-	11	0	0	9	11	33
										
	**AVG**		**38**	**79**		**30**	**61**		**30**	**69**
	**ACC**		57	**82**		**31**	**59**		**30**	**57**

**WoLF PSORT**	ex	78	93	97	7	36	79	1	0	0
	pm	34	41	59	29	59	79	6	83	83
	pe	3	0	0	5	0	0	2	0	0
	er	25	8	40	46	9	54	6	0	50
	go	14	0	7	8	0	0	6	17	17
	ly	4	0	25	-	-	-	-	-	-
	va	-	-	-	11	0	0	9	0	33
										
	**AVG**		24	38		17	35		17	31
	**ACC**		**58**	68		22	51		20	40

The sensitivity (SE) and top three sensitivity (SE3) for each localization are shown. SE3 measures the fraction of correctly predicted proteins within the top three ranked localizations. The corresponding average sensitivity and overall accuracy are listed also, with the top-scoring highlighted in bold. Values based on very few proteins (less than six) are drawn in gray. All measures are given as percentages.

## Conclusion

Our new approach for predicting protein subcellular localization, MultiLoc2, integrates several subpredictors based on the overall amino acid composition, the detection of sorting signals, phylogenetic profiles and GO terms. Compared to the original MultiLoc architecture, the robustness and prediction performance is clearly improved. The different resolutions of MultiLoc2 were compared with current state-of-the-art sequence-based methods using independent datasets.

MultiLoc2-LowRes is specialized for globular proteins and offers kingdom-specific prediction of up to five localizations based on the BaCelLo dataset. On the other hand, MultiLoc2-HighRes is able to deal with membrane proteins and predicts all of the main eukaryotic localizations based on a dataset that consists of a mixture of animal, fungal and plant proteins. In comparison with five other methods, the MultiLoc2 predictors performed better for animal and plant proteins whereas MultiLoc2-LowRes outperforms MultiLoc2-HighRes in general. However, the performance of MultiLoc2-HighRes is remarkable since it is able to predict more localizations than the other tools except for WoLF PSORT. We also simulated the scenario in which no GO term is available for any test proteins, which makes the prediction sequence-based only. The resulting performance of the MultiLoc2 predictors is slightly reduced but still better for animals and plants and comparable for fungi. Therefore, we conclude that the MultiLoc2 approach is very robust and well suited for novel proteins without relevant sequence similarity to annotated proteins but can also benefit from the presence of calculated GO annotation from the sequence using InterProScan.

In a second benchmark study we evaluated the prediction performance of MultiLoc2-HighRes compared to WoLF PSORT for proteins localized in the peroxisomes and in the sublocalizations of the secretory pathway. For all three eukaryotic kingdoms, MultiLoc2-HighRes performs clearly better. In particular, MultiLoc2-HighRes shows much better sensitivity throughout all localizations and yields high robustness. However, the results indicate that the classification in all main eukaryotic localizations is still a challenging task and leaves room for improvement for future work.

The flexible architecture of MultiLoc2 is based on the easily extendable protein profile vector. In the future, this will allow us to integrate more heterogeneous and relevant information to further improve the prediction accuracy. In particular, we plan to investigate in further sequences-based or annotation-based information such as protein-protein interaction and text-terms from PubMed abstracts. Moreover, handling of proteins present in multiple locations is an open challenge.

## Availability and requirements

**• Project name**: MultiLoc2

**• Project home page**: 

**• Operating system(s)**: Linux

**• Programming language**: Python

**• Other requirements**: LIBSVM 2.8 or higher, BLAST 2.2.14 or higher, InterProScan 4.3 or higher (optional)

**• License**: GNU GPL

**• Any restrictions to use by non-academics**: None

## Authors' contributions

All the authors have read and approved the final manuscript.

## Supplementary Material

Additional file 1 **Supplementary Materials**. Supplementary Materials (PDF file) include description of some methodology details such as the NCBI genomes used in PhyloLoc, the number of GO terms used in GOLoc, and an overview of the MultiLoc2-LowRes architecture. In addition, result details are provided including the performance evaluation on the independent datasets without GO terms. Click here for file
