# Supplementary material for: MultiLoc2: integrating phylogeny and Gene Ontology terms improves subcellular protein localization prediction
Source: BMC Bioinformatics. 2009 Sep 1;10:274. doi: 10.1186/1471-2105-10-274 (PMC2745392; doi:10.1186/1471-2105-10-274)
Supplement: Additional file 1 — Supplementary Materials. Supplementary Materials (PDF file) include description of some methodology details such as the NCBI genomes used in PhyloLoc, the number of GO terms used in GOLoc, and an overview of the MultiLoc2-LowRes architecture. In addition, result details are provided including the performance evaluation on the independent datasets without GO terms. [file 1471-2105-10-274-S1.pdf]

Table 1: The number of protein sequences listed for the BacelLo training data set according to each origin and localization.

**Localization**	**Plants**	**Animal**	**Fungi**
chloroplast (ch)	204	-	-
cytoplasm (cy)	58	439	211
mitochondria (mi)	67	188	188
nucleus (nu)	121	1166	711
secretory pathway (SP)	41	804	88
**Total**	**491**	**2597**	**1198**

Supplementary material

Training datasets

The number of protein sequences for each localization in the BacelLo and Höglund training data sets are shown in Tab. 1 and Tab. 2.

Phylogenetic profiles

We downloaded 453 fully sequenced genomes from the National Center for Biotechnology Information (NCBI) ftp site (*ftp.ncbi.nih.gov/genomes*) consisting of 20 eukaryotes, 33 archaea and 400 bacteria. However, only 78 genomes were used for the calculation of phylogenetic profiles. We used all eukaryotic and archaea genomes and selected only the 25 genetically most distant bacteria genomes in order to get an approximately equal distribution of genomes from the three domains of life. We used the same genome subselection procedure as described in Sun *et al.*, 2005. The method uses the NCBI taxonomy information to reconstruct a taxonomy tree and exploits hierarchical information in a top down approach to select a preferably nonredundant set of genomes. The complete set of the genomes used is listed in Tab. 3, Tab. 4 and Tab. 5.

Table 2: The number of protein sequences listed for the Höglund training data set according to each localization.

**Localization**	**Nr**
chloroplast (ch)	449
cytoplasm (cy)	1411
Endoplasmatic Reticulum (er)	198
extracellular space (ex)	843
Golgi apparatus (go)	150
lysosome (ly)	103
mitochondria (mi)	510
nucleus (nu)	837
peroxisome (pe)	157
plasma membrane (pm)	1238
vacuole (va)	63
**Total**	**5959**

Table 3: The selected fully sequenced eukaryotic genomes used for the calculation of phylogenetic profiles.

Kingdom	Taxonomy ID	Organism name
Fungi	4932	Saccharomyces cerevisiae
Fungi	33169	Eremothecium gossypii
Fungi	214684	Cryptococcus neoformans var. neoformans JEC21
Fungi	284590	Kluyveromyces lactis NRRL Y-1140
Fungi	284591	Yarrowia lipolytica CLIB122
Fungi	284592	Debaryomyces hansenii CBS767
Fungi	284593	Candida glabrata CBS 138
Fungi	284812	Schizosaccharomyces pombe 972h-
Fungi	284813	Encephalitozoon cuniculi GB-M1
Metazoa	6239	Caenorhabditis elegans
Metazoa	7227	Drosophila melanogaster
Metazoa	9606	Homo sapiens
Metazoa	10090	Mus musculus
Protista	5693	Trypanosoma cruzi
Protista	35128	Thalassiosira pseudonana
Protista	36329	Plasmodium falciparum 3D7
Protista	280699	Cyanidioschyzon merolae strain 10D
Protista	294381	Entamoeba histolytica HM-1:IMSS
Viridiplantae	3702	Arabidopsis thaliana
Viridiplantae	39947	Oryza sativa Japonica Group

Table 4: The selected fully sequenced archaea genomes used for the calculation of phylogenetic profiles.

**Phylum**	**Taxonomy ID**	**Organism name**
Crenarchaeota	178306	Pyrobaculum aerophilum str. IM2
Crenarchaeota	272557	Aeropyrum pernix K1
Crenarchaeota	273057	Sulfolobus solfataricus P2
Crenarchaeota	273063	Sulfolobus tokodaii str. 7
Crenarchaeota	330779	Sulfolobus acidocaldarius DSM 639
Crenarchaeota	368408	Thermofilum pendens Hrk 5
Crenarchaeota	384616	Pyrobaculum islandicum DSM 4184
Crenarchaeota	415426	Hyperthermus butylicus DSM 5456
Euryarchaeota	64091	Halobacterium sp. NRC-1
Euryarchaeota	69014	Thermococcus kodakarensis KOD1
Euryarchaeota	70601	Pyrococcus horikoshii OT3
Euryarchaeota	186497	Pyrococcus furiosus DSM 3638
Euryarchaeota	187420	Methanothermobacter thermautotrophicus str. Delta H
Euryarchaeota	188937	Methanosarcina acetivorans C2A
Euryarchaeota	190192	Methanopyrus kandleri AV19
Euryarchaeota	192952	Methanosarcina mazei Go1
Euryarchaeota	224325	Archaeoglobus fluorides DSM 4304
Euryarchaeota	243232	Methanocaldococcus jannaschii DSM 2661
Euryarchaeota	259564	Methanococcoides burtonii DSM 6242
Euryarchaeota	263820	Picrophilus torridus DSM 9790
Euryarchaeota	267377	Methanococcus maripaludis S2
Euryarchaeota	269797	Methanosarcina barkeri str. Fusaro
Euryarchaeota	272569	Haloarcula marismortui ATCC 43049
Euryarchaeota	272844	Pyrococcus abyssi GE5
Euryarchaeota	273075	Thermoplasma acidophilum DSM 1728
Euryarchaeota	273116	Thermoplasma volcanium GSS1
Euryarchaeota	323259	Methanospirillum hungatei JF-1
Euryarchaeota	339860	Methanosphaera stadtmanae DSM 3091
Euryarchaeota	348780	Natronomonas pharaonis DSM 2160
Euryarchaeota	349307	Methanosaeta thermophila PT
Euryarchaeota	362976	Haloquadratum walsbyi DSM 16790
Euryarchaeota	410358	Methanocorpusculum labreanum Z
Nanoarchaeota	228908	Nanoarchaeum equitans Kin4-M

Table 5: The selected fully sequenced bacteria genomes used for the calculation of phylogenetic profiles.

**Phylum**	**Taxonomy ID**	**Organism name**
Chloroflexi	243164	Dehalococcoides ethenogenes 195
Chloroflexi	255470	Dehalococcoides sp. CBDB1
Cyanobacteria	1140	Synechococcus elongatus PCC 7942
Cyanobacteria	1148	Synechocystis sp. PCC 6803
Cyanobacteria	59920	Prochlorococcus marinus str. NATL2A
Cyanobacteria	64471	Synechococcus sp. CC9311
Cyanobacteria	103690	Nostoc sp. PCC 7120
Cyanobacteria	197221	Thermosynechococcus elongatus BP-1
Cyanobacteria	203124	Trichodesmium erythraeum IMS101
Cyanobacteria	240292	Anabaena variabilis ATCC 29413
Cyanobacteria	251221	Gloeobacter violaceus PCC 7421
Firmicutes	292459	Symbiobacterium thermophilum IAM 14863
Proteobacteria	60480	Shewanella sp. MR-4
Proteobacteria	62928	Azoarcus sp. BH72
Proteobacteria	62977	Acinetobacter sp. ADP1
Proteobacteria	156889	Magnetococcus sp. MC-1
Proteobacteria	232721	Acidovorax sp. JS42
Proteobacteria	266779	Mesorhizobium sp. BNC1
Proteobacteria	290400	Jannaschia sp. CCS1
Proteobacteria	292414	Silicibacter sp. TM1040
Proteobacteria	296591	Polaromonas sp. JS666
Proteobacteria	326442	Pseudoalteromonas haloplanktis TAC125
Proteobacteria	374463	Baumannia cicadellinicola str. Hc (Homalodisca coagulata)
Proteobacteria	387662	Candidatus Carsonella ruddii PV
Proteobacteria	413404	Candidatus Ruthia magnifica str. Cm (Calyptogena magnifica)

Gene Ontology terms

GOLoc uses only the GO terms that can be observed in the training data since for other GO terms we can not make any statements. In addition, it has the advantage that only a small portion of the over 26000 available GO terms is used. Table 6 shows the number of selected GO terms for every individual predictor. A list of selected GO terms can be obtained from the MultiLoc2 webpage.

Table 6: The selected GO terms for the individual MultiLoc2 predictors.

**MultiLoc2 version**	**Training dataset**	**Number of selected GO terms**
MultiLoc2-LowRes Animals	BaCelLo animals	712
MultiLoc2-LowRes Fungi	BaCelLo fungi	508
MultiLoc2-LowRes Plants	BaCelLo plants	408
MultiLoc2-HighRes Animals	MultiLoc animals	1097
MultiLoc2-HighRes Fungi	MultiLoc fungi	1075
MultiLoc2-HighRes Plants	MultiLoc plants	1155

MultiLoc2-LowRes architecture

The architecture of the animal version of MultiLoc2-LowRes is shown in Fig. 1. Compared with MultiLoc2-HighRes the SVMSA subpredictor is not used because MultiLoc2-LowRes is specialized on globular proteins.

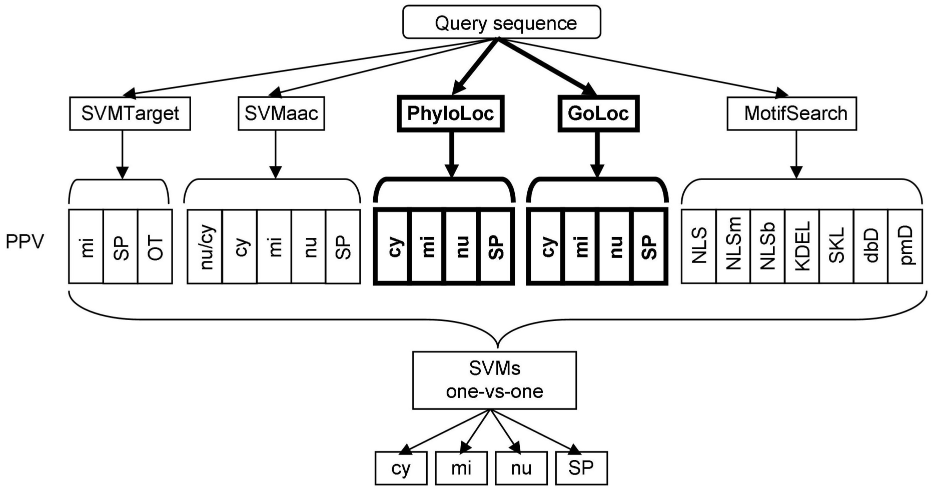

Figure 1: The architecture of MultiLoc2-LowRes (animal version). A query sequence is processed by a first layer of five subprediction methods (SVMTarget, SVMaac, PhyloLoc, GOLoc and MotifSearch). The individual output of the layer one methods are collected in the PPV which enters a second layer of SVMs producing probability estimates for each localization.

Independent test without GO terms

The results of the simulation that no GO terms are available for all proteins of the independent data set are presented in this section. Tab. 7 shows the localization-specific performance results using sensitivity and MCC and Tab. 8 summarizes the overall performances using AVG and ACC.

MultiLoc2-LowRes

The animal prediction performance of MultiLoc2-LowRes is reduced by only one per cent regarding to AVG and ACC when predicting three classes and by two and four percent when predicting four classes. The reason is that more nuclear proteins are wrongly predicted if we discard the GO terms. The fungal prediction performance is almost unchanged which is mainly caused by the fact that on average only 34% of the fungal proteins are annotated

with GO terms by InterProScan. The plant ACCs are decreased from 83% to 80% and from 76% to 71% for the prediction of three classes and four classes respectively. This is caused by the dropping sensitivity of the nuclear proteins (from 91% to 77%). The AVGs are reduced by nine per cent which seems to be a very significant performance lost at the first view. The reason is that the SP sensitivity is reduced from 83% to 50%. Only two SP proteins are additionally wrong predicted if we neglect the GO annotation. However, these two proteins have a large impact on the AVGs because the SP cluster contains only six proteins overall.

MultiLoc2-HighRes

Similar to the MultiLoc2-LowRes, the fungal prediction performance of MultiLoc2-HighRes is almost unchanged. This is the same for the plants in case of the prediction of four classes. The performance reduction by three per cent for the prediction of five plant classes is also moderate. However, very different to MultiLoc2-LowRes, the animal ACCs are reduced by nine percent and 11% respectively. We analyzed the additionally wrong predicted proteins and found out that this was caused by a failure in the clustering procedure performed by the curators of the data set [Casadio *et al.*, 2008]. The nuclear data set contains 56 proteins of the protamine-P1 family. Each protein represents one cluster which biases the prediction towards this over-represented protein class. The reason for the failed clustering could be the relatively short sequences of the proteins between 50 and 60 amino acids. Therefore, we reclustered the nuclear proteins using BLASTClust and 30% sequence identity. Now, the 56 proteins of the protamine-P1 family are clustered and the new number of clusters is 186 for the nuclear proteins and 277 for the nu/cy class. The comparison of the animal results based on the reclustered nuclear proteins delivers only a slightly performance reduction. We also applied BLASTClust on all other localizations and always received either the same number of clusters or a few more which indicates that the described clustering problem did not appear for the remaining classes.

References

Sun,J., Xu,J., Liu,Z., Liu,Q., Zhao,A., Shi,T., Li,Y. (2005) Refined phylogenetic profiles method for predicting protein-protein interactions., *Bioinformatics*, **16**, 3409-15.

Casadio,R., Martelli,P.,L., Pierleoni,A. (2008) The prediction of protein subcellular localization from sequence: a shortcut to functional genome annotation, *Brief Funct Genomic Proteomic.*, **7(1)**, 63-73.

Table 7: Comparison of the localization-specific prediction results of the MultiLoc2 predictors using BacelLo independent dataset (BaCelLo IDS)

**Version**	**Loc**	**Nr**	**ML2-LowRes**		**ML2-LowRes***		**ML2-HighRes**		**ML2-HighRes***	
			SE	MCC	SE	MCC	SE	MCC	SE	MCC
Animals	SP	75	97	0.89	97	0.88	87	0.79	88	0.60
	mi	48	89	0.81	86	0.78	83	0.75	83	0.74
	nu	224	62	0.57	56	0.52	58	0.54	36	0.34
	cy	85	72	0.43	72	0.38	71	0.39	72	0.37
	nu/cy	308	93	0.87	92	0.84	91	0.78	77	0.63
Animals$^+$	SP	75	97	0.89	97	0.88	87	*0.82*	88	*0.80*
	mi	48	89	*0.80*	86	0.78	83	0.75	83	*0.73*
	nu	*186*	*54*	*0.51*	*46*	*0.44*	*52*	*0.50*	*45*	*0.43*
	cy	85	72	*0.41*	72	*0.35*	71	*0.37*	72	*0.35*
	nu/cy	*277*	*92*	*0.85*	*91*	*0.83*	*91*	*0.79*	*89*	*0.77*
Fungi	SP	9	78	0.60	78	0.59	78	0.63	78	0.63
	mi	77	68	0.62	66	0.61	51	0.52	54	0.55
	nu	152	63	0.36	63	0.36	50	0.32	44	0.28
	cy	180	54	0.27	54	0.27	56	0.22	54	0.18
	nu/cy	332	92	0.63	93	0.66	84	0.48	83	0.47
Plants	SP	6	83	0.58	50	0.40	83	0.50	83	0.47
	mi	6	67	0.51	67	0.45	67	0.40	67	0.42
	ch	72	77	0.72	78	0.70	53	0.51	54	0.51
	nu	36	91	0.77	77	0.63	86	0.74	79	0.64
	cy	17	41	0.38	41	0.33	37	0.20	29	0.12
	nu/cy	52	94	0.84	88	0.76	93	0.74	91	0.70

The sensitivity (SE) and Matthews correlation coefficient (MCC) of MultiLoc2 (ML2) are listed for each localization (Loc). The number of clusters (Nr) per localization is also shown. The results for MultiLoc2-LowRes* and MultiLoc2-HighRes* are obtained by simulating that for all test proteins no GO term is available. The Animals$^+$ dataset was obtained by reclustering the nuclear proteins from the original animals dataset. Changes in performance are highlighted in italic.

Table 8: Comparison of the overall performance results of the MultiLoc2 predictors using BacelLo independent dataset (BaCelLo IDS)

**Version**	**Classes**	**ML2-LowRes**	**ML2-LowRes***	**ML2-HighRes**	**ML2-HighRes***
Animals	3	93 (93)	92 (92)	87 (89)	83 (80)
	4	80 (73)	78 (69)	75 (68)	70 (57)
Animals$^+$	3	93 (93)	*91* (92)	87 (89)	*87 (88)*
	4	*78 (70)*	*75 (66)*	*73 (67)*	*72 (64)*
Fungi	3	79 (87)	79 (88)	71 (78)	72 (77)
	4	66 (60)	65 (60)	59 (52)	58 (51)
Plants	4	80 (83)	71 (80)	74 (70)	74 (70)
	5	72 (76)	63 (71)	65 (62)	62 (59)

The average sensitivity and the overall accuracy (in parenthesis) of MultiLoc2 (ML2) for the prediction of three and four classes for animals and fungi and four and five classes for plants are shown. The results for MultiLoc2-LowRes* and MultiLoc2-HighRes* are obtained by simulating that for all test proteins no GO term is available. The Animals$^+$ dataset was obtained by reclustering the nuclear proteins from the original animals dataset. Changes in performance are highlighted in italic.
